# Examining the Cross-Country Differences in the Adverse Childhood Experiences Associated With Men’s Interest in and Perpetration of Technology-Facilitated Child Sexual Exploitation and Abuse

**DOI:** 10.1177/08862605251403610

**Published:** 2026-03-03

**Authors:** Tyson Whitten, Michael Salter, Delanie Woodlock, Ashleigh McFeeters, Inga Vermeulen, Sarah Louise Guthrie, Konstantinos Kosmas Gaitis, Deborah Fry

**Affiliations:** 1 University of New South Wales, Sydney, Australia; 2 University of Edinburgh, Scotland, UK

**Keywords:** adverse childhood experiences, child maltreatment, offenders/perpetrators, sexual abuse

## Abstract

Rates of technology-facilitated child sexual exploitation and abuse (TF-CSEA) have substantially increased over the past decade. Addressing associated factors such as Adverse Childhood Experiences (ACEs) may be critical for large-scale prevention. This study examined the relationship between ACEs and adult men categorised as having (a) no TF-CSEA interest or perpetration, (b) TF-CSEA interest only, and (c) TF-CSEA perpetration. Independent quota-based samples from Australia (*n* = 1,939), the United Kingdom (*n* = 1,506), and the United States (*n* = 1,473) were analysed, with data weighted to be demographically comparable to the male populations. Results show that ACEs were markedly more prevalent among TF-CSEA perpetrators, with proportions higher in the United Kingdom (28.2%–67.8%) and United States (35.9%–64.0%) than in Australia (16.0%–35.6%). Multivariate analyses indicate that emotional abuse (adjsuted OR = 0.52), sexual abuse (aOR = 1.77), and household incarceration (aOR = 2.05) were associated with TF-CSEA interest only, while sexual abuse (aOR = 3.64) and neglect (aOR = 2.27) were associated with TF-CSEA perpetration, relative to no TF-CSEA interest or perpetration, and after adjusting for other ACE types. Furthermore, emotional (aOR = 2.55) and sexual abuse (aOR = 2.10) were more likely for the TF-CSEA perpetration than the interest only group. Interaction effects showed that emotional abuse had a stronger association in the United Kingdom than in Australia, and neglect in the United Kingdom than in the United States. These findings underscore the association between childhood adversity, especially sexual abuse, and TF-CSEA, and highlight the importance of policies addressing ACEs as part of a broader prevention strategy.

## Introduction

Global estimates indicate that more than 300 million children are sexually abused online each year (Childlight – Global Child Safety Institute, 2024). Efforts to identify and respond to technology-facilitated child sexual exploitation and abuse (TF-CSEA) are outpaced by its scale as well as the emerging technologies used by perpetrators to aid offending and avoid detection (Broadhurst, 2019; WeProtect, 2021). Preventing TF-CSEA by identifying and addressing early correlates of offending, specifically Adverse Childhood Experiences (ACEs), may be a more sustainable approach. This study is the first cross-country comparison of the relationship between ACEs and TF-CSEA interest and perpetration among adult men from Australia (*n* = 1,939), the United Kingdom (*n* = 1,506), and the United States (*n* = 1,473). Children are broadly defined as anyone under 18 years of age, aligning with the international standards set by the U.N. Convention on the Rights of the Child. This paper begins with a literature review outlining the relationship between ACEs and child sex offending, as well as reasons for potential cross-country differences. Next, the study methodology is presented followed by the results, which report the between and within-country differences in the prevalence of ACEs and TF-CSEA interest and perpetration, followed by analyses of the independent association between ACEs and TF-CSEA outcomes, with interaction effects highlighting cross-country differences in the strength and direction of these associations. The paper closes with a discussion of the implications of these findings for indicated intervention and broader government policy. To avoid deterministic interpretations, it is important to clarify that although ACEs are associated with increased risk, most individuals who experience them do not engage in TF-CSEA.

## Literature Review

TF-CSEA is defined as the transmission, access, and solicitation of online sexual material that depicts or represents someone under the age of 18 years, is illegal in Australia (Criminal Code Act 1995 (Cth) s. 473.1), the United States (18 U.S.C. § 2252), and United Kingdom (Protection of Children Act 1978, c. 37, s.1). Reports of TF-CSEA, which can include images and videos, AI-generated content, and livestreams, have increased over the last decade (Internet Watch Foundation, 2022), with further spikes associated with children’s online engagement during and following the COVID-19 pandemic (Madigan et al., 2022). The psychological and social consequences of TF-CSEA to victims are substantial (Ali & Paash, 2022; Joleby et al., 2020), costing economies billions of dollars annually (Giles et al., 2024). More so, detecting and prosecuting TF-CSEA offenders is difficult, given the global scale of the issue, and perpetrators’ use of anonymity tools and software to conceal their identity and locations (Broadhurst, 2019; WeProtect, 2021). A public health approach that aims to prevent TF-CSEA by mitigating factors associated with perpetration may be key to reducing this issue at scale (Letourneau et al., 2014).

ACEs are stressful and potentially traumatic events that occur before age 18 (Merrick et al., 2019) and are well-documented correlates of criminal offending (Basto-Pereira et al., 2022). These experiences include various forms of abuse (e.g. physical, emotional, and sexual), neglect (e.g. maltreatment and lack of family support), and household dysfunction (e.g. exposure to domestic violence, parental divorce, and substance abuse). Individuals exposed to these factors early in life are significantly more likely to abuse drugs, experience mental ill-health, and have frequent and persistent interactions with the criminal justice system (Astridge et al., 2023; Hughes et al., 2017; Malvaso et al., 2022). The negative outcomes associated with ACEs appear to be compounded when multiple adverse experiences occur together, leading to a more significant cumulative effect than any single experience (Testa et al., 2022; Van Wert et al., 2017). However, ACEs do not necessarily directly *cause* criminal behaviour. Instead, the consequences of early adversity could accumulate over time, potentially interacting with other idiosyncratic and environmental factors that contribute to the development of criminogenic behaviours and psychopathologies (Perez et al., 2018), including sexual interests and behaviours towards children (Grady et al., 2017).

People who sexually offend against children are significantly more likely to have a history of ACEs, especially sexual abuse, compared to non-sex offenders, the general non-offending population (Kahn et al., 2021; Levenson et al., 2017), and non-offenders with a sexual interest in children (Jahnke et al., 2023; Stephens et al., 2023). The “*sexually abused – sexual abuser hypothesis*” is one prominent explanation for this connection, asserting that childhood sexual abuse is associated with a greater risk of later child sex offending. This hypothesis suggests that mechanisms such as social learning (Felson & Lane, 2009), disrupted psychosexual development (Jespersen et al., 2009), and possible genetic predispositions towards paraphilic interests (Långström et al., 2015) mediate this link. Although sexual abuse often co-occurs with other childhood aversities, this perspective emphasises sexual abuse as the primary driver of child sex offending.

Grady et al. (2017) present an alternative explanation, rooted in Bowlby’s (1973) attachment theory, which argues that it is the cumulative effect of ACEs, rather than sexual abuse alone, that creates pathways to sexually abusive behaviour. Accordingly, ACEs broadly disrupt attachment formation and socioemotional development, undermining the capacity to form secure bonds with caregivers. This disruption fosters insecure attachment styles marked by emotional dysregulation, interpersonal difficulties, and maladaptive coping strategies. These insecure attachments shape internal working models that influence future relational expectations and behaviours, often manifesting as deficits in empathy, intimacy, and self-regulation. These challenges are exacerbated by the neurobiological impacts of ACEs, which can impair stress regulation and hinder healthy brain development, increasing vulnerability to deviant sexual interests and offense-supportive beliefs. Within this framework, sexual offending is conceptualised as a maladaptive response to cumulative psychosocial and neurobiological difficulties.

Building on the link between ACEs and TF-CSEA, research suggests this relationship may be mediated by factors such as deviant sexual interests (e.g. paraphilias involving animals or violence), anxiety, depression, substance misuse, and low social support (Seto et al., 2015; Steene et al., 2023). Demographically, TF-CSEA offenders are more likely to be younger, well educated, have a higher income, and never lived with a partner, relative to contact and non-sex offenders (Babchishin et al., 2015; Faust et al., 2015). Online and offline child sex offenders appear to share several characteristics, including sexual orientation, employment status, mental health issues, and childhood experiences of neglect and domestic violence (Babchishin et al., 2015; Levenson et al., 2017). Online only and those with both online and offline child sex offences also appear comparable in terms of age, income, education, ethnicity, and general psychological profiles. Notably, much of the extant literature focuses on men due to their majority status among child sex offenders, recognising that female child sex offenders, while exhibiting some overlapping patterns, significantly differ in their risk factors, circumstances, motivations, and tactics (Burgess-Proctor et al., 2017).

Current estimates from several community-based samples indicate that around one-in-twenty men have self-reported sexual interests towards children, and one-in-fifty consume TF-CSEA. For example, Dombert et al. (2016) found that 4.1% of German men reported fantasies involving prepubescent children, with 2.4% admitting to consuming child pornography. In the Czech Republic, Bártová et al. (2021) reported that 2.3% and 16.8% of men had sexual interest in prepubescent and pubescent children. Similarly, Joyal and Carpentier (2017) reported that 1.1% of Canadian men expressed sexual interests towards prepubescent children. Seto et al. (2015) noted that among Swedish men aged 17 to 20 years, 4.2% had viewed child pornography, and 9.9% were likely or very likely to be sexually interested in children. Finally, an international online survey (Ó Ciardha et al., 2022) involving 997 men – predominantly from the United Kingdom and United States – revealed that 6.8% would watch child pornography if guaranteed anonymity and no consequences, while 3.3% admitted to having deliberately viewed such material online since turning 18.

Some emerging evidence suggests that the proportion of children who have been the victim of sexual abuse differs across countries, even among culturally similar Western nations (Moody et al., 2018). Although cross-country research on the prevalence of TF-CSEA offenders is sparse, recent findings from the same data used in the current study found that 10.9% of U.S. men, compared to 7.5% of Australian and 7.0% of U.K. men, had engaged in TF-CSEA or had sexual conversations with a child online (Salter et al., 2023). Additionally, a higher proportion of U.S. men (8.5%) reported that they would have sexual contact with a child aged 14 years or younger if they could avoid detection, compared to their counterparts in Australia (5.8%) and the United Kingdom (4.2%). This suggests that the United States may have a higher proportion of men who harbour sexual interests and behaviours towards children than other Western English-speaking countries, a finding supported by another cross-country comparison (Ó Ciardha et al., 2022). Moreover, this aligns with data showing that the United States generally has higher rates of ACEs than other English-speaking nations (Moody et al., 2018; Stoltenborgh et al., 2015).

Variations in the prevalence of TF-CSEA offenders across Australia, the United Kingdom, and United States may also be attributed to differences in each country’s legislative frameworks governing online surveillance and monitoring. Both Australia and the United Kingdom have federal legislation requiring that proactive protection mechanisms be built into online platforms and service provision to ensure the protection of children online (Beatriz & Schertel Mendes, 2023; Cooray et al., 2023). It also requires the timely removal of child sexual abuse material from platforms once a notice has been sent or the material has been located by mandated proactive searching. The United States lacks a similar piece of federal legislation and hosts the majority of known CSAM URLs globally (Internet Watch Foundation, 2022).

Understanding the link between ACEs and TF-CSEA perpetration is key for early prevention and intervention strategies, as it provides insights into the developmental factors associated with the risk of offending. Differentiating ACEs associated with non-perpetrators who have an interest in TF-CSEA is also important, as this research can help identify the protective factors that mitigate the transition from interest to perpetration. Exploring how these associations vary across countries can also help elucidate the role of cultural, legal, and social contexts in shaping behaviours and risks. This comparative research can lead to globally informed, country-specific practices that optimise the effectiveness of prevention efforts, support services, and legal frameworks in protecting children online. Importantly, effective prevention of TF-CSEA relies on accurate information about the characteristics of undetected offenders. However, most existing research is based on small forensic or convenience samples, with few large-scale studies that are predominantly focused on European populations and lack detailed data on offenders’ online behaviour, social networks, and experiences of childhood adversity (Savoie et al., 2021). There is also a lack of international comparisons of TF-CSEA offenders, suggesting a presumed uniformity in offender profiles across different countries.

The current study uses data from a large multi-jurisdictional study of men from Australia, the United Kingdom, and United States to examine the cross-country differences in the relationship between individual and cumulative ACEs and three categories of TF-CSEA involvement: (a) no interest or perpetration, (b) interest without perpetration, and (c) perpetration. The analysis began by examining the prevalence of ACEs within each TF-CSEA category across the three countries, followed by pooled analyses exploring the correlation between cumulative ACE scores and TF-CSEA outcomes. Logistic regression models were then developed to assess the associations between ACEs and TF-CSEA categories, adjusting for demographic factors and country. These models considered all ACE types to identify their unique contributions and were further extended to include interaction effects, examining cross-country variations in these relationships. Models were adjusted for age, education, income, and marital status to account for cross-country demographic differences associated with TF-CSEA perpetration (Babchishin et al., 2015).

## Methods

### Data

An online survey was conducted examining the prevalence and factors associated with men’s sexual attitudes, feelings, and behaviours towards children. Data were drawn from three samples of men aged 18 years or over, quota-matched to be comparable to the Australian, U.K., and U.S. male populations in terms of age, residential region, annual household income, and educational attainment. Survey recruitment and administration were conducted by CloudResearch (https://www.cloudresearch.com), an online research panel company with access to an international pool of over 100 million participants. The survey used Prime Panels, which aggregates various market research platforms with opt-in participants profiled on numerous variables. Invitations were sent based on demographic profiles, and participants received compensation determined by their respective platforms. As Prime Panels sources participants from multiple platforms, the total number of individuals invited or who accessed the study could not be determined. The survey was reviewed by a project advisory group, which included representatives from law enforcement, financial intelligence units, government departments, and mental health support services. Surveys were administered from November to December 2022. Ethical approval for this study was provided by the University of New South Wales (HC220317) prior to data collection.

The study invitation described the research as a “survey about men’s attitudes and behaviours relating to online child abuse.” It stated that eligible participants were men aged 18 or over and explained that the survey would cover topics including demographic background, online behaviours, mental health, pornography use, childhood abuse, and attitudes toward child sexual abuse. The invitation emphasised anonymity and confidentiality and noted that participation was voluntary and would take approximately 15 to 20 min. CloudResearch uses its Sentry® system to screen participants for inattentiveness, dishonesty, duplicate entries, VPN use, and bot activity. It also includes built-in attention checks during survey routing, excluding those who fail. In addition, a mid-survey attention check was used: “*The color test you are about to take part in is very simple. When asked to select your favourite colour, you must select ‘green’. This is an attention check. What is your favourite colour?*”.

Of the 7,343 individuals who consented to participate (Australia = 2,703; United Kingdom = 2,243; United States = 2,397), 6,577 completed the survey (retention rates: Australia 92.2%, United Kingdom 87.4%, United States 88.6%). After excluding participants who were not male at birth (*n* = 198), did not identify as male (*n* = 178), failed attention checks (*n* = 1,506), or admit dishonest responses (*n* = 115), and removing 68 with missing key demographic data, the final analytical sample included 4,918 participants (Australia = 1,939; United Kingdom = 1,506; United States = 1,473). For a detailed description of the study methodology, see Whitten et al (I﻿n press).

### Measures

#### TF-CSEA Interest and Perpetration

Current interest in TF-CSEA was measured using two items adapted from Malamuth’s (1989) attraction to sexual aggression scale (also see: Wurtele et al., 2014; Seto et al., 2015). Participants responded either *yes*, *no*, *maybe*, or *unsure* to the following: (1) “*if you could be sure that you were anonymous online, would you watch pornographic material of people below the age of 18*?” (*n* = 267); and (2) “*if you were offered a webcam sex show of someone under the age of 18, would you watch it?*” (*n* = 277). A dichotomous variable was created indicating if participants responded yes to either question (Spearman-Brown split-half coefficient = 0.46).

Three items devised by the researchers were used to measure TF-CSEA perpetration. Men responded *yes* or *no* according to if they had engaged in any of the following behaviours while aged 18 years or over: (a) “*I knowingly and deliberately viewed pornographic material containing people below the age of 18*” (*n* = 169); (b) “*I have engaged in a sexually explicit webcam interaction with a person below the age of 18*” (*n* = 117), and; (c) “*I have paid for online sexual interactions, images or videos involving a person below the age of 18*” (*n* = 134). A composite variable was created, indicating engagement in one or more of these behaviours (Spearman-Brown split-half coefficient = 0.48). Participants were then grouped into three non-overlapping categories, designated (a) no TF-CSEA interest or perpetration, (b) TF-CSEA interest only, and (c) TF-CSEA perpetration.

#### Adverse Childhood Experiences

Based on Felitti et al.’s (1998) questionnaire (ACE-Q), participants reported (1 = *yes*, 0 = *no*) if they had experienced any of the following adversities prior to the age of 18: (a) emotional abuse (*did a parent or other adult in the household often or very often swear at you, insult you, put you down, or humiliate you, or act in a way that made you afraid that you might be physically hurt?*); (b) physical abuse (*did a parent or other adult in the household often or very often push, grab, slap, or throw something at you, or ever hit you so hard that you had marks or were injured?*); (c) sexual abuse (*an adult or person at least five years older than you ever touch or fondle you or have you touch their body in a sexual way, or attempt or have oral, anal, or vaginal intercourse with you*?); (d) low family support (*did you often or very often feel that no one in your family loved you or thought you were important or special, or your family didn’t look out for each other, feel close to each other, or support each other?*); (e) neglect (*did you often or very often feel that you didn’t have enough to eat, had to wear dirty clothes, and had no one to protect you, or your parents were too drunk or high to take care of you or take you to the doctor if you needed it*?); (f) parental divorce (*were your parents ever separated or divorced*?); (g) domestic violence (*was your mother or stepmother often or very often pushed, grabbed, slapped, or had something thrown at her, or sometimes, often, or very often kicked, bitten, hit with a fist, or hit with something hard, or ever repeatedly hit for at least a few minutes or threatened with a gun or knife?*); (h) household drug abuse (*did you live with anyone who was a problem drinker or alcoholic or who used street drugs*?); (i) household mental illness (*was a household member depressed or mentally ill, or did a household member attempt suicide?*), and; (j) household member incarcerated (*did a household member ever go to prison*?). Responses were summed to create a cumulative ACE score (range 0–10; α = .83). The psychometric properties of the ACE-Q have been extensively studied, often exceeding the minimum thresholds for acceptable reliability and validity (for a recent meta-analysis, see Lyons et al., 2023).

#### Demographic Characteristics

Age was measured as six bandings (18–24 years, 25–34 years, 35–44 years, 45–54 years, 55–64 years, and 65 years or older). Educational attainment was categorised as (1) did not finish high school, (2) completed high school or equivalent, (3) vocational degree, diploma, or equivalent, and (4) bachelor’s degree or higher. Annual household income before taxes was coded as (1) low (*less than AUD$50,000, £20,000 or US$25,000*), (2) middle (*between AUD$50,000–$149,999, £20,000–£59,999, or US$25,000–$99,999*), and (3) high (*equal to or more than AUD$150,000, £60,000, or US$100,000*). Current marital status was designated (1) married, (2) de facto relationship, (3) widowed, separated, or divorced, and (4) never married.

### Analytical Strategy

Descriptive statistics with 99% Confidence Intervals (CI) were presented for the demographic characteristics, TF-CSEA categories, and ACEs separately for the Australian, U.K., and U.S. samples. The overlap with specific ACE types was also examined. Also calculated was the pooled prevalence of men in the TF-CSEA interest only and TF-CSEA perpetration groups within each cumulative ACE score (range 0–10). The strength and direction of the correlation between cumulative ACE scores and these categories, relative to the no TF-CSEA interest or perpetration group, were calculated using the two-tailed point-biserial correlation (*r*_pb_). Next presented were the prevalence of ACEs within the (a) no TF-CSEA interest or perpetration, (b) TF-CSEA interest only, and (c) TF-CSEA perpetration groups, stratified by country. Within-country differences were detected using the adjusted *F* statistic variant of the second-order Rao-Scott chi-square test. Between country differences were identified using the *F* statistic derived from the Wald chi-square test.

A series of logistic regression analyses were conducted to examine the adjusted association between ACEs and (model 1) TF-CSEA interest only relative to no TF-CSEA interest or perpetration, (model 2) TF-CSEA perpetration relative to no TF-CSEA interest or perpetration, and (model 3) TF-CSEA perpetration relative to TF-CSEA interest only. Data were pooled (*N* = 4,918) to minimise overfitting and type 2 errors. Adjusted models control for all ACE types to identify their independent contribution to the outcome, as well as demographic characteristics and country. Odds ratios (OR) and 99% confidence intervals (99% CIs) were reported as measures of effect size and precision of the association between ACEs and outcomes. Cross-country interaction effects (β[se]) were modelled separately from the main effect models and indicate whether the strength or direction of the associations significantly differs between countries. Separate regression models were conducted examining the association between cumulative ACE scores and TF-CSEA interest and perpetration, adjusted for demographic factors and country only (specific ACE types were not included in these models).

Data were weighted using iterative proportional fitting, calibrating the weight of each participant until the sample and population distribution were comparable in terms of age, annual household income, race, educational attainment, marital status, and workforce participation based on benchmark categories sourced from each country’s respective 2021 census of men (see Whitten et al (In press)). The median weight of each participant was 0.81 for the Australian sample, 0.92 for the U.K. sample, and 0.84 for the U.S. sample. The weighted scores for 27 participants exceeded the median weight plus six times the interquartile range; these cases were truncated to reduce mean squared errors of the outcome estimates (Battaglia et al., 2009). Analyses were conducted using a complex sample design (Zou et al., 2019), which adjusted the standard errors for poststratification weights (Lumley, 2004). No missing data were present. To account for multiple comparisons, results were considered statistically significant if *p* < .01 or 99% CI do not cross 1 (Bender & Lange, 2001). Analyses were conducted using IBM SPSS version 29 (IBM Corp, 2022).

## Results

### Descriptive Statistics

Table 1 presents the descriptive statistics for the Australian (*n* = 1,939), U.K. (*n* = 1,506), and U.S. (*n* = 1,473) samples of adult men. There was no significant difference in age-bracket proportions across the three countries (*F*(10, 4917) = 1.34, *p* = .20). There were significant cross-country differences in annual household income (*F*(4, 4923) = 74.70, *p* < .001) and educational attainment (*F*(6, 4921) = 51.15, *p* < .001), with non-overlapping 99% CIs indicating that a significantly higher proportion of men from the U.S. (33.8%) than from Australian (24.4%) and the U.K. (11.5%) were in the high income band, while a lower proportion of U.S. men (31.2%) than Australian (38.9%) and U.K. men (41.5%) had a bachelor’s degree or higher. Marital status also significantly differed across countries (*F*(6, 4921) = 5.03, *p* < .001), with the proportion of married men being slightly lower in Australia (44.8%) than the United Kingdom (51.6%) and United States (49.8%).

**Table 1.** Descriptive Statistics and 99% CI for Three Samples of Men From Australia, the United Kingdom, and United States.

Measures	Australia (*n* = 1,939), %	United Kingdom (*n* = 1,506), %	United States (*n* = 1,473), %
Age group			
18–24 years	15.3 [13.4, 17.3]	11.0 [9.1, 13.2]	12.0 [10.1, 14.2]
25–34 years	17.6 [15.8, 19.7]	17.1 [15.0, 19.4]	18.0 [15.9, 20.4]
35–44 years	16.9 [15.0, 19.1]	16.5 [14.5, 18.8]	17.4 [15.4, 19.8]
45–54 years	15.6 [13.6, 17.8]	17.1 [15.1, 19.3]	16.0 [14.1, 18.2]
55–-64 years	14.4 [12.5, 16.5]	16.1 [14.2, 18.3]	16.5 [14.5, 18.8]
65+ years	20.1 [18.1, 22.4]	22.2 [19.8, 24.8]	19.9 [17.8, 22.2]
Annual household income			
Low income	28.9 [25.4, 32.6]	19.0 [16.2, 22.1]	17.4 [14.5, 20.6]
Middle income	46.7 [43.2, 50.2]	69.5 [66.1, 72.7]	48.8 [45.0, 52.6]
High income	24.4 [21.5, 27.7]	11.5 [9.9, 13.3]	33.8 [30.2, 37.7]
Educational attainment			
Did not finish high school	15.8 [13.0, 19.1]	9.1 [6.8, 11.9]	11.5 [8.5, 15.3]
Completed high school	23.6 [20.9, 26.5]	42.6 [38.9, 46.4]	45.9 [42.2, 49.6]
Vocational degree, diploma, or equivalent	21.7 [19.0, 24.8]	6.8 [5.6, 8.3]	11.5 [9.3, 14.1]
Bachelor’s degree or higher	38.9 [35.4, 42.4]	41.5 [37.7, 45.4]	31.2 [27.9, 34.6]
Marital status			
Married	44.8 [41.2, 48.3]	51.6 [47.8, 55.5]	49.8 [46.0, 53.6]
De facto relationship	13.5 [11.5, 15.9]	11.1 [9.3, 13.2]	9.7 [7.6, 12.2]
Widowed, separated, or divorced	11.0 [8.8, 13.8]	9.8 [7.5, 12.7]	13.3 [10.9, 16.0]
Never married	30.7 [27.4, 34.2]	27.5 [24.1, 31.1]	27.3 [24.0, 30.8]
TF-CSEA interest and perpetration categories			
No TF-CSEA interest or perpetration	88.9 [86.4, 90.9]	89.0 [86.2, 91.2]	86.0 [83.1, 88.5]
TF-CSEA interest only	6.4 [4.8, 8.4]	6.4 [4.6, 8.7]	4.9 [3.5, 6.9]
TF-CSEA perpetration	4.8 [3.6, 6.4]	4.7 [3.3, 6.6]	9.1 [7.1, 11.6]
Adverse Childhood Experiences			
Emotional abuse	26.3 [23.2, 29.6]	23.8 [20.6, 27.2]	33.7 [30.2, 37.4]
Physical abuse	22.4 [19.5, 25.5]	21.5 [18.4, 24.9]	26.1 [22.9, 29.6]
Sexual abuse	10.2 [8.2, 12.5]	10.5 [8.3, 13.1]	16.0 [13.4, 19.0]
Low family support	20.9 [18.1, 23.9]	20.5 [17.6, 23.8]	27.0 [23.8, 30.5]
Neglect	9.0 [7.3, 11.2]	9.2 [7.3, 11.6]	16.1 [13.4, 19.1]
Parental divorce	27.9 [24.8, 31.2]	25.9 [22.6, 29.5]	37.4 [33.8, 41.2]
Domestic violence	9.7 [7.9, 11.9]	9.3 [7.3, 11.9]	15.7 [13.1, 18.8]
Household drug abuse	17.4 [15.0, 20.2]	14.0 [11.5, 16.9]	27.8 [24.5, 31.4]
Household mental illness	16.4 [14.1, 19.1]	14.4 [11.9, 17.3]	18.9 [16.0, 22.1]
Household member in jail	5.7 [4.3, 7.5]	7.2 [5.5, 9.5]	16.8 [14.0, 19.9]
Mean cumulative ACEs	1.66 [1.51, 1.81]	1.56 [1.39, 1.73]	2.36 [2.14, 2.57]

The prevalence of TF-CSEA interest only did not significantly differ between Australia (6.4%), the United Kingdom (6.4%), and the United States (4.9%) based on the global test (*F*(4, 4923) = 1.31, *p* = .27). In contrast, the prevalence of TF-CSEA perpetration significantly differed across countries (*F*(4, 4923) = 13.20, *p* < .001). Post hoc comparisons using 99% CI indicated that the United States (9.1%) had a significantly higher prevalence than both Australia (4.8%) and the United Kingdom (4.7%).

Global tests indicate significant between-country differences in emotional abuse (*F*(2, 4652) = 14.60, *p* < .001), physical abuse (*F*(2, 4652) = 3.68, *p* = .03), sexual abuse (*F*(2, 4652) = 11.36, *p* < .001), low family support (*F*(2, 4652) = 8.73, *p* < .001), neglect (*F*(2, 4652) = 19.06, *p* < .001), parental divorce (*F*(2, 4652) = 19.60, *p* < .001), domestic violence (*F*(2, 4652) = 14.39, *p* < .001), household drug abuse (*F*(2, 4652) = 36.80, *p* < .001), household mental illness (*F*(2, 4652) = 4.07, *p* = .02), and a household member in jail (*F*(2, 4652) = 48.56, *p* < .001). Men from the United States reported significantly higher rates of most ACEs compared to those from Australia and the United Kingdom as indicated by non-overlapping 99% CIs. Similarly, the total number of ACEs differed significantly across countries (*F*(2, 4652) = 36.44, *p* < .001), with non-overlapping 99% CIs indicating that U.S. men reported significantly more ACEs on average than their Australian and U.K. counterparts.^1^

**Figure 1.**
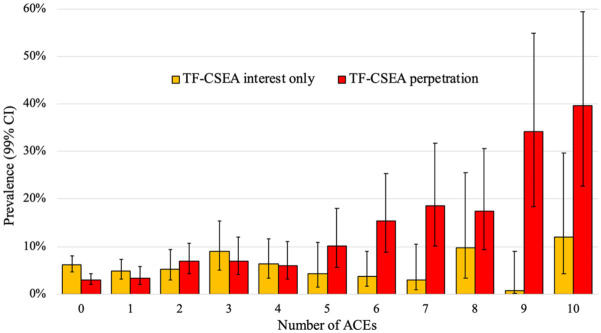
Prevalence (99% CI) of TF-CSEA interest only and TF-CSEA perpetration by number of ACEs. *Note.* TF-CSEA = technology-facilitated child sexual exploitation and abuse; ACEs = Adverse Childhood Experiences.

Figure 1 presents the pooled (*N* = 4,918) prevalence of TF-CSEA interest only and TF-CSEA perpetration by cumulative ACE score. There is a significant upward trend in the pooled data between the number of ACEs and TF-CSEA perpetration (*r*_pb_ = .23, *p* < .001). This positive correlation is separately present for the Australian (*r*_pb_ = .13, *p* < .001), U.K. (*r*_pb_ = .28, *p* < .001), and U.S. samples (*r*_pb_ = .26, *p* < .001). The relationship between the number of ACEs and TF-CSEA interest only was non-significant for the pooled sample (*r*_pb_ = .01, *p* = .72) and for the Australian (*r*_pb_ < .00, *p* = .96), U.K. (*r*_pb_ = .02, *p* = .48), and U.S. (*r*_pb_ = .01, *p* = .60) samples, separately.

Table 2 presents the row proportions of men with overlapping ACE types for the pooled sample (*n* = 4,918). Abuse-related ACEs co-occurred the most: physical and emotional abuse frequently overlap (81.9% of those with physical also reported emotional abuse; 68.5% vice versa), and both showed high overlap with low family support and domestic violence. Neglect also exhibited a broad overlap (e.g. 76.7% with physical abuse; 70.4% with low family support). Childhood sexual abuse co-occurred with other ACEs in roughly one third of cases, especially domestic violence (46%), neglect (42.9%), and household member in jail (42.1%). The lowest overlap was for household member in jail, ranging from 20.5% of men who experienced emotional abuse to 35.0% of those who experienced neglect.

**Table 2.** Row Proportions of Men With Overlapping (%) ACE Types (*n* = 4,918).

ACE		1	2	3	4	5	6	7	8	9	10
1	Emotional abuse (*n* = 1,365)	–	68.5%	29.5%	60.5%	30.9%	50.7%	32.0%	43.2%	35.3%	20.5%
2	Physical abuse (*n* = 1,142)	81.9%	–	32.0%	58.7%	34.7%	50.6%	36.8%	44.1%	36.2%	22.5%
3	Sexual abuse (*n* = 590)	68.1%	62.0%	–	54.2%	40.0%	52.5%	43.7%	49.2%	41.2%	33.3%
4	Low family support (*n* = 1,112)	74.3%	60.3%	28.8%	–	34.9%	51.2%	32.6%	44.8%	38.5%	21.9%
5	Neglect (*n* = 551)	76.7%	72.0%	42.9%	70.4%	–	58.4%	48.3%	59.7%	47.7%	35.0%
6	Parental divorce (*n* = 1,482)	46.7%	39.0%	20.9%	38.4%	21.7%	–	25.6%	40.6%	30.7%	21.5%
7	Domestic violence (*n* = 561)	77.9%	75.0%	46.0%	64.7%	47.4%	67.6%	–	62.9%	50.4%	35.7%
8	Household drug abuse (*n* = 958)	61.5%	52.6%	30.2%	52.0%	34.3%	62.8%	36.7%	–	44.6%	29.2%
9	Household mental illness (*n* = 813)	59.3%	50.9%	29.9%	52.6%	32.3%	56.0%	34.8%	52.5%	–	29.6%
10	Household member in jail (*n* = 466)	60.1%	55.2%	42.1%	52.1%	41.4%	68.3%	42.9%	60.0%	51.6%	–

*Note.* ACE = Adverse Childhood Experience.

### Cross-Country Prevalence of ACEs Within TF-CSEA Categories

Table 3 presents the country-specific prevalence of ACEs within the (a) no TF-CSEA interest or perpetration, (b) TF-CSEA interest only, and (c) TF-CSEA perpetration groups. Across each sample, within-country differences consistently demonstrate that a significantly (*p* < .01) higher proportion of men in the TF-CSEA perpetration group than the no TF-CSEA interest or perpetration group experienced sexual abuse, neglect, domestic violence, household drug abuse, and a household member in jail. Within-country differences for the United Kingdom and United States also show that this group had significantly higher proportions of emotional abuse, physical abuse, low family support, and household mental illness. Compared to the TF-CSEA interest only group, the TF-CSEA perpetration group had a significantly higher prevalence of domestic violence across all countries, whereas the prevalence of emotional abuse, physical abuse, and sexual abuse were significantly higher in the United Kingdom and United States, while neglect was higher in Australia and the United Kingdom. Finally, across all countries, the mean number of cumulative ACEs was significantly higher for the TF-CSEA perpetration than the TF-CSEA interest only and no TF-CSEA interest or perpetration group.

**Table 3.** Row Prevalence (99% CI) of ACEs and (1) No TF-CSEA Interest or Perpetration, (2) TF-CSEA Interest Only, and (3) TF-CSEA Perpetration, Stratified by Country (Between Group and Country Comparisons).

ACEs	Groups	Australia (*N* = 1,939)	United Kingdom (*N* = 1,506)	United States (*N* = 1,473)	Between country
Emotional abuse	1	26.3% [23.0%, 29.9%]	21.6% [18.4%, 25.1%]	30.9% [27.3%, 34.8%]	*F*(2, 4652) = 10.79, *p* < .001
	2	19.8% [11.0%, 33.0%]	22.2% [12.0%, 37.5%]	26.5% [14.3%, 43.8%]	*F*(2, 4652) = 0.44, *p* = .64
	3	34.9% [22.6%, 49.6%]	67.8% [50.6%, 81.3%]	64.0% [50.8%, 75.3%]	*F*(2, 4652) = 9.79, *p* < .001
Within country		*F*(2, 3858) = 2.38, *p* = .09	*F*(2, 2996) = 30.58, *p* < .001	*F*(2, 2949) = 23.31, *p* < .001	
Physical abuse	1	22.0% [19.0%, 25.4%]	19.3% [16.2%, 22.8%]	22.9% [19.6%, 26.5%]	*F*(2, 4652) = 1.99, *p* = .14
	2	19.9% [11.0%, 33.3%]	24.2% [13.3%, 40.0%]	23.4% [12.0%, 40.8%]	*F*(2, 4652) = 0.25, *p* = .78
	3	32.3% [20.2%, 47.2%]	59.5% [42.1%, 74.8%]	58.5% [45.7%, 70.2%]	*F*(2, 4652) = 7.16, *p* < .001
Within country		*F*(2, 3862) = 2.16, *p* = .12	*F*(2, 2999) = 23.97, *p* < .001	*F*(2, 2952) = 30.85, *p* < .001	
Sexual abuse	1	8.8% [6.8%, 11.2%]	7.9% [5.9%, 10.4%]	12.2% [9.8%, 15.1%]	*F*(2, 4652) = 5.65, *p* = .004
	2	15.1% [7.2%, 29.2%]	14.1% [6.2%, 28.9%]	19.3% [9.0%, 36.7%]	*F*(2, 4652) = 0.31, *p* = .73
	3	29.6% [18.2%, 44.4%]	54.8% [37.5%, 71.1%]	50.3% [37.8%, 62.8%]	*F*(2, 4652) = 5.17, *p* = .006
Within country		*F*(2, 3804) = 15.79, *p* < .001	*F*(2, 3003) = 57.63, *p* < .001	*F*(2, 2952) = 49.89, *p* < .001	
Low family support	1	20.8% [17.9%, 24.1%]	18.7% [15.8%, 22.0%]	24.6% [21.3%, 28.3%]	*F*(2, 4652) = 5.20, *p* = .006
	2	14.8% [7.4%, 27.4%]	27.2% [15.3%, 43.6%]	27.0% [14.7%, 44.2%]	*F*(2, 4652) = 2.27, *p* = .11
	3	30.2% [19.0%, 44.5%]	46.5% [29.9%, 64.0%]	50.1% [37.6%, 62.6%]	*F*(2, 4652) = 3.70, *p* = .025
Within country		*F*(2, 3844) = 3.00, *p* = .05	*F*(2, 2998) = 12.20, *p* < .001	*F*(2, 2950) = 15.61, *p* < .001	
Neglect	1	7.8% [6.1%, 10.0%]	7.5% [5.7%, 9.8%]	12.9% [10.3%, 16.0%]	*F*(2, 4652) = 11.21, *p* < .001
	2	11.8% [5.3%, 24.2%]	5.7% [1.8%, 16.9%]	23.7% [12.1%, 41.4%]	*F*(2, 4652) = 4.73, *p* = .009
	3	28.0% [17.1%, 42.4%]	46.5% [29.8%, 64.1%]	41.8% [30.0%, 54.6%]	*F*(2, 4652) = 2.69, *p* = .07
Within country		*F*(2, 3843) = 17.16, *p* < .001	*F*(2, 2990) = 49.69, *p* < .001	*F*(2, 2952) = 29.65, *p* < .001	
Parental divorce	1	27.9% [24.6%, 31.5%]	25.4% [21.9%, 29.1%]	36.8% [32.9%, 40.8%]	*F*(2, 4652) = 16.20, *p* < .001
	2	26.5% [15.6%, 41.3%]	26.2% [15.0%, 41.8%]	38.1% [22.5%, 56.5%]	*F*(2, 4652) = 1.22, *p* = .30
	3	29.0% [17.4%, 44.1%]	35.4% [20.6%, 53.7%]	43.0% [31.0%, 56.0%]	*F*(2, 4652) = 1.80, *p* = .17
Within country		*F*(2, 3883) = 0.17, *p* = .94	*F*(2, 3008) = 3.53, *p* = .28	*F*(2, 2949) = 2.03, *p* = .48	
Domestic violence	1	8.7% [6.8%, 11.0%]	8.0% [6.0%, 10.5%]	13.0% [10.5%, 16.1%]	*F*(2, 4652) = 8.17, *p* < .001
	2	8.9% [4.0%, 18.5%]	7.7% [2.9%, 19.2%]	18.0% [8.1%, 35.3%]	*F*(2, 4652)=2.28, *p* = .104
	3	29.6% [17.9%, 44.9%]	37.0% [21.7%, 55.5%]	40.0% [28.3%, 53.1%]	*F*(2, 4652) = 0.98, *p* = .38
Within country		*F*(2, 3861) = 18.62, *p* < .001	*F*(2, 2976) = 26.37, *p* < .001	*F*(2, 2952) = 25.14, *p* < .001	
Household drug abuse	1	16.4% [13.9%, 19.2%]	12.6% [10.1%, 15.6%]	26.3% [22.8%, 30.1%]	*F*(2, 4652) = 32.93, *p* < .001
	2	18.1% [9.4%, 32.1%]	20.0% [9.9%, 36.2%]	26.7% [13.5%, 46.1%]	*F*(2, 4652) = 0.67, *p* = .51
	3	35.6% [23.0%, 50.7%]	32.2% [17.8%, 50.9%]	43.0% [31.0%, 55.9%]	*F*(2, 4652) = 1.00, *p* = .37
Within country		*F*(2, 3834) = 8.10, *p* < .001	*F*(2, 3002) = 8.28, *p* < .001	*F*(2, 2937) = 5.92, *p* = .003	
Household mental illness	1	15.7% [13.2%, 18.5%]	13.5% [11.0%, 16.5%]	16.8% [13.9%, 20.1%]	*F*(2, 4652) = 2.18, *p* = .11
	2	16.9% [8.6%, 30.7%]	15.2% [6.7%, 30.7%]	21.5% [9.3%, 42.4%]	*F*(2, 4652) = 0.36, *p* = .70
	3	29.4% [17.9%, 44.2%]	30.3% [16.7%, 48.4%]	37.0% [25.7%, 50.0%]	*F*(2, 4652) = 0.69, *p* = .50
Within country		*F*(2, 3826) = 4.34, *p* = .014	*F*(2, 2994) = 5.39, *p* = .005	*F*(2, 2893) = 10.59, *p* < .001	
Household member in jail	1	4.6% [3.4%, 6.3%]	6.0% [4.3%, 8.3%]	14.1% [11.4%, 17.4%]	*F*(2, 4652) = 37.63, *p* < .001
	2	13.0% [5.3%, 28.6%]	9.1% [3.4%, 22.2%]	27.6% [15.2%, 44.8%]	*F*(2, 4652) = 11.04, *p* < .001
	3	16.0% [7.8%, 29.9%]	28.2% [15.3%, 46.1%]	35.9% [24.7%, 48.9%]	*F*(2, 4652) = 12.63, *p* < .001
Within country		*F*(2, 3705) = 10.82, *p* < .001	*F*(2, 3008) = 18.88, *p* < .001	*F*(2, 2949) = 18.23, *p* < .001	
Mean cumulative ACEs	1	1.59 [1.43, 1.75]	1.40 [1.24, 1.57]	2.10 [1.89, 2.31]	*F*(2, 4652) = 23.47, *p* < .001
	2	1.65 [0.98, 2.31]	1.72 [0.99, 2.44]	2.52 [1.50, 3.54]	*F*(2, 4652) = 1.83, *p* = .16
	3	2.95 [1.93, 3.96]	4.38 [3.35, 5.42]	4.64 [3.74, 5.54]	*F*(2, 4652) = 5.66, *p* = .004
Within country		*F*(2, 4652) = 5.80, *p* = .003	*F*(2, 4652) = 27.17, *p* < .001	*F*(2, 4652) = 25.25, *p* < .001	

*Note.* ACEs = Adverse Childhood Experiences; TF-CSEA = technology-facilitated child sexual exploitation and abuse.

Significant differences between countries indicate that the proportion of those in the TF-CSEA perpetration group who experienced emotional abuse, physical abuse, and sexual abuse was almost twice as high for the United Kingdom and United States than for Australia. For the TF-CSEA interest only group, the proportion who experienced neglect was around two-to-four-times greater for the United States than Australia and the United Kingdom. Furthermore, among those in the no TF-CSEA interest or perpetration group, men from the United States had significantly higher prevalences of almost all ACE types compared to those from Australia and the United Kingdom. The average number of ACEs experienced by those in the TF-CSEA perpetration group was significantly higher for the United Kingdom and United States than for Australia.

### Associations Between ACEs and TF-CSEA Categories

Table 4 presents the results of the demographic and country-adjusted logistic regression models examining the association between ACEs and (model 1) TF-CSEA interest only relative to no TF-CSEA interest or perpetration, (model 2) TF-CSEA perpetration relative to no TF-CSEA interest or perpetration, and (model 3) TF-CSEA perpetration relative to TF-CSEA interest only. Also included are cross-country interaction effects modelled separately from the main effects. Although all ACEs were included in the multivariate models, there was no evidence of multicollinearity (Variance Inflation Factor range = 1.07–2.18; Tolerance range = 0.46–0.93).

**Table 4.** Adjusted Main Effects (OR [99% CI]) and Cross-Country Interaction Effects (β [se]) of the Association Between ACEs and (Model 1) TF-CSEA Interest Only Relative to No TF-CSEA Interest or Perpetration, (Model 2) TF-CSEA Perpetration Relative to No TF-CSEA Interest or Perpetration, and (Model 3) TF-CSEA Perpetration Relative to TF-CSEA Interest Only (*n* = 4,918).

ACES	Model	Main effects	Interaction effects (β [se])		
		OR [99% CI]	Australia vs. United States	United Kingdom vs. United States	United Kingdom vs. Australia
Emotional abuse	1	0.52 [0.27, 0.99]	−.06 (0.41)	.18 (0.44)	.24 (0.42)
	2	1.32 [0.72, 2.42]	−.43 (0.36)	.76 (0.38)	1.19 (0.39)*
	3	2.55 [1.09, 5.96]	−.37 (0.52)	.58 (0.55)	.95 (0.55)
Physical abuse	1	1.15 [0.62, 2.16]	−.02 (0.43)	.24 (0.44)	.26 (0.42)
	2	1.19 [0.65, 2.17]	−.42 (0.36)	.43 (0.38)	.85 (0.40)
	3	1.03 [0.45, 2.38]	−.41 (0.54)	.18 (0.56)	.59 (0.55)
Sexual abuse	1	1.77 [1.00, 3.17]	.13 (0.49)	−.05 (0.53)	−.19 (0.52)
	2	3.64 [2.27, 5.82]	−.17 (0.37)	.54 (0.39)	.65 (0.43)
	3	2.10 [1.02, 4.31]	−.25 (0.59)	.59 (0.62)	.84 (0.62)
Low family support	1	1.08 [0.64, 1.82]	−.39 (0.44)	.35 (0.43)	.74 (0.44)
	2	0.85 [0.49, 1.46]	.08 (0.36)	.42 (0.38)	.35 (0.40)
	3	0.79 [0.38, 1.62]	.47 (0.55)	.07 (0.55)	−.40 (0.57)
Neglect	1	1.29 [0.69, 2.40]	−.14 (0.49)	−1.13 (0.60)	−.99 (0.60)
	2	2.27 [1.31, 3.94]	.37 (0.38)	.59 (0.39)	.22 (0.44)
	3	1.77 [0.80, 3.89]	.31 (0.58)	1.75 (0.67)*	1.21 (0.71)
Parental divorce	1	0.82 [0.51, 1.32]	.08 (0.41)	.11 (0.43)	.12 (0.39)
	2	0.62 [0.37, 1.03]	−.03 (0.38)	.22 (0.42)	.45 (0.42)
	3	0.75 [0.38, 1.46]	−.11 (0.54)	.11 (0.58)	.34 (0.55)
Domestic violence	1	0.79 [0.39, 1.63]	−.13 (0.51)	−.54 (0.57)	−.40 (0.56)
	2	1.63 [0.89, 2.99]	.40 (0.40)	.07 (0.41)	−.33 (0.45)
	3	2.01 [0.84, 5.03]	.54 (0.62)	.61 (0.67)	.07 (0.68)
Household drug abuse	1	1.17 [0.63, 2.18]	.25 (0.46)	.47 (0.49)	.22 (0.46)
	2	1.04 [0.59, 1.84]	.67 (0.37)	.16 (0.42)	.05 (0.36)
	3	0.89 [0.39, 2.02)	.42 (0.57)	−.31 (0.61)	−.74 (0.60)
Household mental illness	1	0.92 [0.51, 1.66)	−.01 (0.53)	−.15 (0.55)	−.14 (0.48)
	2	0.95 [0.57, 1.56)	.49 (0.38)	.16 (0.41)	−.33 (0.44)
	3	1.03 [0.49, 2.16)	.49 (0.62)	.31 (0.66)	−.19 (0.62)
Household member in jail	1	2.05 [1.05, 4.02)	.38 (0.51)	−.44 (0.55)	−.82 (0.60)
	2	1.67 [0.94, 2.96)	.13 (0.48)	.31 (0.46)	.18 (0.57)
	3	0.81 [0.35, 1.87)	−.25 (0.65)	.75 (0.68)	1.00 (0.76)

*Note.* Adjusted for ACEs, country, age, educational attainment, household income, and marital status. Main and interaction effects are modelled separately. ACEs = Adverse Childhood Experiences.

* *p* < .01.

The first model indicates that the odds of TF-CSEA interest only were 1.92 (aOR = 0.52; 99% CI [0.27, 0.99]) times lower for emotional abuse, and 1.77 [1.00, 3.17] and 2.05 [1.05, 4.02] times higher for sexual abuse and household incarceration, respectively, relative to no TF-CSEA interest or perpetration. The second main effects model demonstrates that the odds of TF-CSEA perpetration compared to no TF-CSEA interest or perpetration were 3.64 [2.27, 5.82] times greater for sexual abuse and 2.27 [1.31, 3.94] times greater for neglect. Interaction effects indicate that the association for emotional abuse was significantly greater for the United Kingdom than for Australia (β = 1.19 [*se* = 0.39], *p* < .01). The absence of a significant main effect for emotional abuse suggests a cross-over interaction, where the direction of the association differed between the United Kingdom and other countries. Model 3 shows that the odds of TF-CSEA perpetration compared to TF-CSEA interest only were 2.55 [1.09, 5.96] times greater for emotional abuse, and 2.21 [1.02, 4.31] times greater for sexual abuse. There was also a significant crossover interaction indicating that neglect was associated with higher odds of TF-CSEA in the U.K. relative to the U.S. (β = 1.75 [*se* = 0.67], *p* < .01).

Additional Regression models were calculated for cumulative ACE scores, adjusted for demographic factors and country, but not individual ACE types. Results indicate that each additional ACE was associated with 1.33 times (99% CI [1.25, 1.41]) higher odds of TF-CSEA perpetration, relative to the no TF-CSEA interest or perpetration group, and 1.30 times [1.17, 1.44] higher odds relative to the TF-CSEA interest only group. Cumulative ACEs were not significantly associated with the TF-CSEA interest only group compared to the no TF-CSEA interest or perpetration group (aOR = 1.03 [0.94, 1.11]). No significant cross-country interaction effects were found.

A post hoc simulation-based power analysis was conducted in RStudio (v. 4.4.2) using the *nnet* and *dplyr* packages to assess whether interaction analyses had sufficient statistical power. Data were simulated for Australian (*n* = 1,939), United Kingdom (*n* = 1,506), and United States (*n* = 1,473) men, with outcome probabilities defined across the three TF-CSEA categories based on observed distributions. The simulation included 14 covariates assumed to have an average effect of β = .40. The analysis specified the minimum detectable effect size at 80% power and allowed the relationship between ACEs and the outcome to vary by country. Across 1,000 simulations, power to detect a significant country × ACE interaction was estimated by comparing a full model (with the interaction) to a reduced model (main effects only) using likelihood ratio tests. Results indicated that the minimum detectable effect size α = .01 was β = .63 (corresponding to an aOR of 1.88). Reducing the average effect of covariates to β = .10 resulted in a minimum detectable effect size of β = .41 (aOR = 1.50).

### Sensitivity Analyses

Additional analyses were conducted to account for the possibility that younger TF-CSEA perpetrators (aged 18–24 years) engaged in consensual online behaviours. Supplemental Table 1 presents the pooled proportions of TF-CSEA interest and perpetration categories by age bracket. Men aged under 45 years were overrepresented in the TF-CSEA interest only and perpetration groups, relative to the no interest or perpetration category. Those aged 18 to 24 years comprised 26.4% (99% CI [18.7%, 35.8%]) of the TF-CSEA interest only group, but only 15.3% [9.6%, 23.5%] of the TF-CSEA perpetration group. Overlapping confidence intervals indicate that the proportion of TF-CSEA perpetrators aged 18 to 24 years did not significantly differ from the proportion of men with no TF-CSEA interest or perpetration (11.9% [10.4%, 13.6%]). Notably, almost twice as many perpetrators were aged 25 to 34 years (28.9% [21.9%, 37.1%]) than 18 to 24 years.

Supplemental Table 2 presents the recalculated adjusted main and cross-country interaction effects limited to men aged 25 years and older (*n* = 4,280). The pattern of results was broadly similar to Table 3, albeit with wider confidence intervals and larger standard errors due to the reduction in statistical power. Notably, sexual abuse was no longer significantly associated with TF-CSEA perpetration relative to TF-CSEA interest only, and there was no significant interaction between neglect and the U.K. relative to the U.S.

## Discussion

This is the first study to identify and compare the association between ACEs and TF-CSEA interest and perpetration across three large samples of men from Australia, the United Kingdom, and United States. Prevalence estimates demonstrate that all ACE types were disproportionately more common among men who perpetrated TF-CSEA, whereas the prevalence of ACEs among men who report TF-CSEA interest only was broadly similar to those who had no TF-CSEA interest or perpetration, with the exception of sexual abuse and household incarceration. Regression models indicated that emotional abuse was associated with lower odds of TF-CSEA interest only, while sexual abuse and household incarceration were associated with higher odds, compared to those with no interest or perpetration of TF-CSEA. Likewise, sexual abuse and neglect were associated with higher odds of TF-CSEA perpetration compared to the no interest and perpetration group, while emotional and sexual abuse were associated with greater odds of perpetration compared to the interest only group. Separate analyses also indicated that a higher number of childhood adversities in general increased the odds of perpetration, but not interest. The sparsity of cross-country interaction effects suggests that the link between these ACEs and TF-CSEA may be generalisable across western nations, highlighting the potential universal applicability of prevention and intervention strategies.

The findings of this study provide several important insights that advance knowledge on the aetiology of TF-CSEA perpetration. Notably, childhood sexual abuse was independently associated with TF-CSEA interest and perpetration. This is consistent with the “sexually abused – sexual abuser hypothesis,” which suggests that early sexual abuse may disrupt psychosexual development and foster maladaptive behaviours, including sexual offending (Felson & Lane, 2009; Jespersen et al., 2009). Nonetheless, cumulative ACE scores were not significantly associated with TF-CSEA interest without perpetration, indicating that non-offending individuals with an interest in TF-CSEA may not experience the same compounded adversities as perpetrators. This is consistent with Grady et al. (2017) theory that early cumulative adversity disrupts attachment and socioemotional development. These disruptions can lead to emotional dysregulation, maladaptive coping, and deficits in empathy, amplifying the risk of acting on deviant sexual interests. This potentially suggests that compounded ACEs are associated with a greater risk of psychosocial and neurobiological vulnerabilities, distinguishing perpetrators from non-offending individuals with similar interests.

The finding that childhood sexual abuse was independently associated with TF-CSEA, while each additional ACE was linked to incrementally higher odds of perpetration, suggests two complementary processes. Sexual abuse may confer a specific vulnerability – potentially through trauma-related cognitions, psychosexual learning, or offense-supportive scripts – that distinguishes both interest and perpetration from no involvement. At the same time, cumulative adversity appears to operate as a general risk amplifier that increases the likelihood of translating interest into action. Importantly, this pattern is not deterministic; although sexual abuse is a salient risk marker, many individuals with this history do not offend, particularly when broader adversities are absent or when protective factors (e.g. supportive relationships, stable environments, timely treatment) are present. Applied to the TF-CSEA interest only outcome, the same logic holds: childhood sexual abuse functions as a specific risk marker for the emergence of such interests, while household incarceration – which potentially indexes caregiver loss, instability, stigma, and antisocial modelling – could be understood as a contextual amplifier whose association likely reflects a broader family environment that shapes sexual scripts and weakens inhibitory controls. Taken together, sexual abuse marks specific vulnerability and cumulative ACEs (or markers of broad adversity such as household incarceration) amplify risk, with outcomes contingent on the balance of adversity and protective factors.

Cross-country differences further contextualise these findings. Specifically, TF-CSEA perpetrators in the United States and United Kingdom reported higher rates of ACEs, including emotional, physical, and sexual abuse, as well as neglect and low family support, compared to their Australian counterparts. These disparities likely reflect systemic differences in child protection policies and access to mental health resources. For example, in Australia, child protection is managed by state and territory governments with a focus on prevention and viewed as a public health issue, aiming to create safe environments, and integrating services across sectors for holistic support (Wise, 2017). These policies often emphasise early intervention and comprehensive support for families in crisis, which could reduce the incidence of childhood adversities that may lead to the development of harmful behaviours in adulthood. Alternatively, the U.K.’s child protection system, which is decentralised across its four nations, prioritises the identification and protection of at-risk children supported by a legislative framework that mandates safeguarding actions and sets organisational guidelines (NSPCC Learning, 2024). In contrast, the U.S. operates under a federal system with state-specific implementations, emphasising procedural responses to abuse reports, and heavily involving legal processes in child welfare decisions (Mignon, 2016).

Additionally, Australia may have more accessible and effective mental health care systems for men at risk. Australia’s mental health services are primarily funded through Medicare, which provides subsidised access to a broad range of mental health services. This universal coverage contrasts with the United States, where mental health services often depend on private health insurance, leading to significant disparities in access and affordability (Coombs et al., 2021). Moreover, although mental health services in the United Kingdom are covered by the National Health Service (NHS), there are often long waiting times and variability in service availability (House of Commons, 2023). Furthermore, Australia’s focus on community-based mental health programmes, such as the Headspace centres for youth mental health, represents a proactive approach to treatment and prevention that is only beginning to be explored in the United Kingdom and the United States (Rickwood et al., 2019).

The extant literature suggests that non-offending men who have sexual feelings towards children are less likely than those who sexually offend against children, but more likely than the general population, to have a history of ACEs (Jahnke et al., 2023; Stephens et al., 2023). These findings, which are often based on contact offenders, are somewhat consistent with our estimates, showing that sexual abuse and household incarceration uniquely predicted men with TF-CSEA interest only. Nonetheless, the number of childhood adversities experienced by this group did not distinguish them from those with no TF-CSEA interest or perpetration. This appears consistent with evidence indicating that sexual interest alone is not a sufficient motivator for offending (Cohen et al., 2018); instead, acting on these interests requires a degree of psychosocial and behavioural dysfunction that can develop following accumulative exposure to ACEs (Grady et al., 2017; Swaminath et al., 2023).

The findings of this study highlight several implications and recommendations for addressing TF-CSEA. First, a public health approach focusing on reducing the incidence and impact of ACEs, especially sexual abuse, is likely to be critical to prevent future offending. Universal and targeted interventions (e.g. parent training, family support services, and early childhood education) are recommended to mitigate risks early in life. Routine screening for ACEs within childhood and adolescent healthcare is also important for identifying at-risk individuals and provide timely interventions to address the effects of ACEs before they potentially manifest as harmful behaviours. Second, public education campaigns ought to raise awareness of the signs of child abuse and its long-term consequences. Strengthening child protection systems is equally important for improving real-time safeguarding, early responses to abuse, and interventions to address behavioural and maladaptive coping strategies. Finally, increasing awareness and access to support services for men seeking confidential help for concerning sexual thoughts or behaviours involving children is necessary. Programmes like Stop It Now (www.stopitnow.org), which operate across Australia, the United Kingdom, and the United States, play an important role in preventing child sexual abuse and should be supported and expanded.

Although this study found ACEs to be associated with TF-CSEA perpetration, most individuals who experienced such trauma and adversity did not go on to offend. In the current study, 85.8% (99% CI [83.7%, 87.6%]) of men with one or more ACE, and 69.6% [63.6%, 75.0%] of those who experienced childhood sexual abuse, reported no interest in or perpetration of TF-CSEA. This recognition helps avoid deterministic interpretations, emphasising that ACEs are not destiny. Hence, broader individual, social, and situational factors must also be considered to understand the development of sexual interest and offending towards children.

The key strength of this study is its multi-jurisdictional design that provides a broad view of the prevalence and correlates of TF-CSEA among men in Australia, the United Kingdom, and the United States. This design improves the generalisability of findings across different cultural and legal contexts, which is critical for developing effective prevention strategies. However, the study design also contains several limitations that must be considered. The reliance on self-reported data could introduce desirability or recall bias, given the sensitive nature of the survey. The cross-sectional design of the study also limits the ability to establish the temporal ordering between ACEs and sexual feelings and behaviours towards children; longitudinal studies would be more definitive in determining the direction of these relationships. The measure of TF-CSEA perpetration may capture instances of consensual online behaviours between young adults and older teenagers, potentially inflating perpetration rates.

Several additional methodological limitations should also be noted. The items used to create TF-CSEA categories had relatively low internal consistency, suggesting that the behaviours captured may reflect related but distinct constructs. Furthermore, the psychometric properties of these measures have not been fully validated, and measurement invariance across countries were not formally assessed. While the items reference behaviours that are clearly defined in law across the countries, differences in interpretation or response patterns cannot be ruled out. The study was also not optimally powered to detect small interaction effects, potentially limiting the detection of more subtle cross-country differences in the association between ACE exposure and TF-CSEA outcomes. Replication in independent samples will be important for assessing the stability and generalisability of these findings. Finally, the sample included only men, limiting insights into female-perpetrated TF-CSEA, an area that remains underexplored in the literature.

## Conclusion

This is the first study to examine and compare the link between TF-CSEA and ACEs among men in Australia, the United Kingdom, and the United States. Our findings contribute to the growing evidence base demonstrating that childhood adversity is a key correlate for TF-CSEA; policies acknowledging the widespread and lasting effects of ACEs are essential, and efforts to prevent the abuse and neglect of children and strengthen child protection systems could have a flow on effect that prevents future TF-CSEA by men. This study also highlighted the differences between men who perpetrate TF-CSEA and those who report having sexual interest only, with findings suggesting there is a difference between these groups, which can inform targeted prevention programming for both boys and men. Moving forward, further research should include longitudinal studies to confirm these associations and expand the focus to include female offenders to develop a more inclusive understanding of TF-CSEA perpetration.

## Supplemental Material

sj-docx-1-jiv-10.1177_08862605251403610 – Supplemental material for Examining the Cross-Country Differences in the Adverse Childhood Experiences Associated With Men’s Interest in and Perpetration of Technology-Facilitated Child Sexual Exploitation and Abuse Supplemental material, sj-docx-1-jiv-10.1177_08862605251403610 for Examining the Cross-Country Differences in the Adverse Childhood Experiences Associated With Men’s Interest in and Perpetration of Technology-Facilitated Child Sexual Exploitation and Abuse by Tyson Whitten, Michael Salter, Delanie Woodlock, Ashleigh McFeeters, Inga Vermeulen, Sarah Louise Guthrie, Konstantinos Kosmas Gaitis and Deborah Fry in Journal of Interpersonal Violence

## Notes

1. The higher rates of ACEs among U.S. men are consistent with prior research on retrospective self-reports. For example, a systematic review by Moody et al. (2018) found the median prevalence of self-reported childhood sexual abuse (*n* studies = 56), physical abuse (*n* studies = 40), and neglect (*n* studies = 8) among North American men to be 14.1% (IQR = 4.3%–21.0%), 24.3% (IQR = 14.1%–32.1%), and 16.6% (IQR = 9.7%–36.6%), respectively.
