# Supplementary material for: Examining the Cross-Country Differences in the Adverse Childhood Experiences Associated With Men’s Interest in and Perpetration of Technology-Facilitated Child Sexual Exploitation and Abuse
Source: J Interpers Violence. 2026 Mar 3;41(7-8):1570–99. doi: 10.1177/08862605251403610 (PMC12960769; doi:10.1177/08862605251403610)
Supplement: sj-docx-1-jiv-10.1177_08862605251403610 – Supplemental material for Examining the Cross-Country Differences in the Adverse Childhood Experiences Associated With Men’s Interest in and Perpetration of Technology-Facilitated Child Sexual Exploitation and Abuse [file sj-docx-1-jiv-10.1177_08862605251403610.docx]

| Supplementary table 1. Pooled proportions (99% CI) of TF-CSEA interest and perpetration categories by age bracket (n = 4,918). | | | |
| --- | --- | --- | --- |
| Age group | No TF-CSEA interest or perpetration  (n = 4,330) | TF-CSEA interest only  (n = 292) | TF-CSEA perpetration  (n = 297) |
| 18-24 years | 11.9% (10.4% - 13.6%) | 26.4% (18.7% - 35.8%) | 15.3% (9.6% - 23.5%) |
| 25-34 years | 16.6% (15.0% - 18.4%) | 20.6% (14.1% - 29.0%) | 28.9% (21.9% - 37.1%) |
| 35-44 years | 16.6% (15.0% - 18.3%) | 17.2% (11.7% - 24.6%) | 22.2% (15.9% - 30.1%) |
| 45-54 years | 17.1% (15.4% - 19.0%) | 8.7% (4.8% - 15.2%) | 9.5% (5.5% - 15.7%) |
| 55-64 years | 16.3% (14.7% - 18.1%) | 10.0% (5.6% - 17.4%) | 9.7% (5.5% - 16.6%) |
| 65+ years | 21.4% (19.5% - 23.3%) | 17.3% (11.2% - 25.4%) | 14.4% (10.2% - 20.0%) |

| Supplementary table 2. Adjusted main effects (OR [99% CI]) and cross-country interaction effects (*β* (se)) of the association between ACEs and (model 1) TF-CSEA interest only relative to no TF-CSEA interest or perpetration, (model 2) TF-CSEA perpetration relative to no TF-CSEA interest or perpetration, and (model 3) TF-CSEA perpetration relative to TF-CSEA interest only, limited to men aged 25 years and older (n = 4,280). | | | | | |
| --- | --- | --- | --- | --- | --- |
| ACES | Model | Main effects | Interaction effects (*β* (se)) | | |
|  |  | OR (99% CI) | AU. vs. U.S.A | U.K. vs. U.S.A. | U.K. vs. AU. |
| Emotional abuse | 1 | 0.50 (0.23 – 1.07) | -0.23 (0.46) | 0.03 (0.49) | 0.26 (0.48) |
|  | 2 | 1.60 (0.83 – 3.08) | -0.56 (0.39) | 0.63 (0.41) | 1.20 (0.41)^*^ |
|  | 3 | 3.22 (1.35 – 7.66) | -0.29 (0.58) | 0.79 (0.65) | 1.09 (0.66) |
| Physical  abuse | 1 | 1.45 (0.71 – 2.98) | -0.07 (0.47) | 0.37 (0.48) | 0.43 (0.46) |
|  | 2 | 1.17 (0.60 – 2.27) | -0.47 (0.39) | 0.24 (0.40) | 0.70 (0.43) |
|  | 3 | 0.97 (0.41 – 2.31) | -0.38 (0.64) | 0.15 (0.65) | 0.53 (0.64) |
| Sexual abuse | 1 | 2.13 (1.14 – 3.99) | -0.24 (0.53) | -0.40 (0.56) | -0.17 (0.56) |
|  | 2 | 3.54 (2.13 – 5.87) | -0.17 (0.40) | 0.49 (0.40) | 0.66 (0.46) |
|  | 3 | 1.72 (0.75 – 3.98) | 0.32 (0.66) | 1.10 (0.70) | 0.78 (0.70) |
| Low family support | 1 | 0.77 (0.43 – 1.39) | -0.29 (0.50) | 0.43 (0.50) | 0.71 (0.49) |
|  | 2 | 0.89 (0.50 – 1.59) | -0.17 (0.38) | 0.29 (0.40) | 0.46 (0.42) |
|  | 3 | 0.91 (0.38 – 2.20) | 0.05 (0.65) | 0.36 (0.68) | 0.31 (0.67) |
| Neglect | 1 | 1.22 (0.60 – 2.47) | -0.48 (0.56) | -1.08 (0.66) | -0.60 (0.68) |
|  | 2 | 2.26 (1.25 – 4.09) | -0.02 (0.40) | 0.40 (0.41) | 0.42 (0.46) |
|  | 3 | 1.43 (0.55 – 3.73) | 0.48 (0.71) | 1.40 (0.84) | 0.92 (0.88) |
| Parental divorce | 1 | 0.84 (0.49 – 1.44) | -0.17 (0.46) | 0.27 (0.49) | 0.45 (0.44) |
|  | 2 | 0.62 (0.36 – 1.08) | 0.06 (0.41) | 0.31 (0.44) | 0.25 (0.48) |
|  | 3 | 0.85 (0.38 – 1.90) | 0.26 (0.64) | 0.42 (0.71) | 0.15 (0.68) |
| Domestic violence | 1 | 0.83 (0.38 – 1.79) | -0.30 (0.58) | -0.18 (0.63) | 0.13 (0.60) |
|  | 2 | 1.32 (0.69 – 2.53) | 0.29 (0.44) | -0.07 (0.44) | -0.36 (0.49) |
|  | 3 | 1.84 (0.69 – 4.94) | 0.86 (0.77) | 0.12 (0.76) | -0.74 (0.80) |
| Household  drug abuse | 1 | 1.32 (0.65 – 2.69) | -0.09 (0.50) | 0.29 (0.52) | 0.39 (0.49) |
|  | 2 | 0.94 (0.52 – 1.70) | 0.84 (0.39) | 0.12 (0.46) | -0.72 (0.49) |
|  | 3 | 0.75 (0.33 – 1.69) | 0.84 (0.63) | -0.05 (0.71) | -0.89 (0.70) |
| Household mental illness | 1 | 0.91 (0.45 – 1.85) | -0.57 (0.57) | -0.90 (0.61) | -0.33 (0.55) |
|  | 2 | 0.98 (0.57 – 1.69) | 0.62 (0.41) | 0.13 (0.43) | -0.49 (0.47) |
|  | 3 | 0.96 (0.40 – 2.35) | 1.07 (0.68) | 0.96 (0.83) | -0.11 (0.84) |
| Household member in jail | 1 | 2.43 (1.11 – 5.33) | -0.12 (0.57) | -0.63 (0.62) | -0.51 (0.68) |
|  | 2 | 1.78 (0.96 – 3.30) | -0.06 (0.53) | 0.41 (0.48) | 0.47 (0.61) |
|  | 3 | 0.87 (0.34 – 2.21) | 0.41 (0.70) | 1.29 (0.85) | 0.88 (0.84) |
| Adjusted for ACEs, country, age, educational attainment, household income, and marital status. Main and interaction effects modelled separately. | | | | | |
| ^*^*p* < .01 | | | | | |
